# Extremely low-frequency electromagnetic fields facilitate both osteoblast and osteoclast activity through Wnt/β-catenin signaling in the zebrafish scale

**DOI:** 10.3389/fcell.2024.1340089

**Published:** 2024-02-07

**Authors:** Jingjing Kobayashi-Sun, Isao Kobayashi, Makoto Kashima, Jun Hirayama, Makiko Kakikawa, Sotoshi Yamada, Nobuo Suzuki

**Affiliations:** ^1^ Department of Clinical Engineering, Faculty of Health Science, Komatsu University, Komatsu, Ishikawa, Japan; ^2^ Faculty of Biological Science and Technology, Institute of Science and Engineering, Kanazawa University, Kanazawa, Ishikawa, Japan; ^3^ Department of Biomolecular Science, Faculty of Science, Toho University, Funabashi, Chiba, Japan; ^4^ Department of Production System Engineering and Sciences, Faculty of Production System Engineering and Sciences, Komatsu University, Komatsu, Ishikawa, Japan; ^5^ Noto Marine Laboratory, Institute of Nature and Environmental Technology, Kanazawa University, Kanazawa, Ishikawa, Japan

**Keywords:** electromagnetic fields, osteoblast, osteoclast, zebrafish scale, Wnt/*β*-catenin signaling

## Abstract

Electromagnetic fields (EMFs) have received widespread attention as effective, noninvasive, and safe therapies across a range of clinical applications for bone disorders. However, due to the various frequencies of devices, their effects on tissues/cells are vary, which has been a bottleneck in understanding the effects of EMFs on bone tissue. Here, we developed an *in vivo* model system using zebrafish scales to investigate the effects of extremely low-frequency EMFs (ELF-EMFs) on fracture healing. Exposure to 10 millitesla (mT) of ELF-EMFs at 60 Hz increased the number of both osteoblasts and osteoclasts in the fractured scale, whereas 3 or 30 mT did not. Gene expression analysis revealed that exposure to 10 mT ELF-EMFs upregulated *wnt10b* and Wnt target genes in the fractured scale. Moreover, *β*-catenin expression was enhanced by ELF-EMFs predominantly at the fracture site of the zebrafish scale. Inhibition of Wnt/*β*-catenin signaling by IWR-1-endo treatment reduced both osteoblasts and osteoclasts in the fractured scale exposed to ELF-EMFs. These results suggest that ELF-EMFs promote both osteoblast and osteoclast activity through activation of Wnt/*β*-catenin signaling in fracture healing. Our data provide *in vivo* evidence that ELF-EMFs generated with a widely used commercial AC power supply have a facilitative effect on fracture healing.

## Introduction

Bone is continuously metabolized by 2 cell types, osteoblasts and osteoclasts. Osteoblasts produce a bone matrix composed of type I collagen, which is further calcified to form bone. In contrast, osteoclasts dissolve calcified bone by acidification and further degrade the bone matrix by collagenase. Bone tissue is thus maintained by the balance between osteoblasts and osteoclasts, and the imbalanced activity of these 2 cell types causes various bone disorders (Boyle et al., 2003; Raggatt and Partridge, 2010; Chen et al., 2018). In recent years, fish scales have been recognized as an attractive model for bone research. Fish scales are the exoskeleton that covers the body surface and are homologous organs to mammalian bone, and show many similarities to mammalian bone, such as the co-existence of osteoblasts and osteoclasts, response to hormones, and calcium metabolism (Pasqualetti et al., 2012; Aman et al., 2018; Hirayama et al., 2023). We recently reported a unique fracture healing model using the zebrafish scale of two transgenic lines, *trap:GFP* and *osterix:mCherry*, which can label osteoclasts and osteoblasts with GFP and mCherry, respectively. Upon induction of fracture stimulation by partial scale cutting, we observed that both *trap:GFP* ^+^ osteoclasts and *osterix:mCherry* ^+^ osteoblasts accumulated at the fracture site. Combined with live-imaging analysis, this model allows us to trace the dynamics of osteoclasts and osteoblasts in the process of fracture healing (Kobayashi-Sun et al., 2020a).

Electromagnetic fields (EMFs) have been shown to accelerate fracture healing and increase bone mass, and have received extensive attention as effective, noninvasive, and safe therapies in a variety of clinical applications for bone disorders since the 1970s (Bassett et al., 1974; Assiotis et al., 2012). EMFs are generally classified into three types: static, sinusoidal, and pulsed EMFs (Consales et al., 2012; Huang et al., 2023). Static and sinusoidal EMFs are generated by direct current (DC) and alternating current (AC) flowing through the electric coil, respectively. In contrast, pulsed electromagnetic fields (PEMFs) are generated using low-frequency magnetic fields with specific waveforms and amplitudes. PEMFs have been studied most extensively for their effects on bone tissue. In a mouse model of osteoporosis, exposure to PEMFs has been shown to increase alkaline phosphatase (ALP) activity and type I collagen production by osteoblasts and increase bone mass (Esposito et al., 2012; Di Bartolomeo et al., 2022). On the other hand, PEMFs are inhibitory to osteoclasts and thus may be effective in treating osteoporosis (Zhou et al., 2013; Pi et al., 2019). Although the effect of EMFs varies greatly with frequency, different manufacturers produce different generators of PEMFs with different waveforms and amplitudes, resulting in large variations in the frequency of PEMFs. Thus, the use of PEMFs makes it difficult to determine optimal EMF conditions in bone repair, which has been a bottleneck in therapeutic applications of EMFs.

Depending on the frequency, sinusoidal EMFs are subdivided into three types: extremely low- (∼300 Hz), intermediate- (300 Hz ∼ 10 MHz), and high-frequency EMFs (10 MHz ∼ 300 GHz). Extremely low-frequency EMFs (ELF-EMFs) have been shown to produce a stimulating effect at the tissue and cellular level via magnetically induced currents in the body, termed “eddy currents,” which are thought to have physiological and clinical effects without carcinogenic or side effects (Markov and Colbert, 2000). ELF-EMF therapy may also be effective in treating a variety of orthopedic conditions (Caliogna et al., 2021). However, there are still few comprehensive evaluations in the existing literature to determine the optimal frequency, intensity of EMFs, and processing time for bone stimulation. Moreover, little is also known about the molecular mechanisms underlying the effect of ELF-EMFs on bone tissue.

In the present study, we investigated the effects of ELF-EMFs at 60 Hz on osteoblasts and osteoclasts at the cellular and molecular level using zebrafish scales. Our data showed that exposure to 10 mT of ELF-EMFs increased the number of osteoblasts and osteoclasts in the fractured scale, while 3 or 30 mT did not. Exposure to 10 mT ELF-EMFs upregulated *wnt10b* expression and enhanced Wnt/*β*-catenin signaling in the fractured scale. Inhibition of Wnt/*β*-catenin signaling by treatment with IWR-1-endo reduced both osteoblasts and osteoclasts in the fractured scale. Thus, we provide *in vivo* evidence that under optimal conditions of exposure, ELF-EMFs promote both osteoblast and osteoclast activity under fracture stress.

## Materials and methods

### Maintenance of zebrafish and goldfish and fracture stimulation

Zebrafish transgenic lines of *Tg(trap:GFP-CAAX)* ^*ou2031Tg*^ and *Tg(osterix:Lifeact-mCherry)* ^*ou2032Tg*^ (Kobayashi-Sun et al., 2020a) were raised in a circulating aquarium system (AQUA) at 28.5°C in a 14/10 h light/dark cycle and maintained according to standard protocols (Westerfield, 1995). Goldfish are maintained in a circulating aquarium at 25°C. All experiments were performed in accordance with a protocol approved by the Committee on Animal Experimentation of Kanazawa University. For fracture stimulation, adult zebrafish were anesthetized in system water containing 0.02% tricaine (Sigma), and the epidermis portion of the scales was cut approximately 400 μm in length with fine scissors under a stereo microscope (Axiozoom V16, Zeiss).

### ELF-EMF exposure

The device for generating ELF-EMFs was newly constructed for the present study. Sinusoidal EMFs were generated by excitation of a unidirectional coil in orthogonal direction at a constant frequency of 60 Hz (Miyakawa et al., 2001). There is a space at the center of the device where a ring-shaped tank can be placed, allowing zebrafish or extracted scales to be uniformly exposed to EMFs.

For exposure of goldfish scales to ELF-EMFs, goldfish were anesthetized with 0.01% tricaine, and goldfish scales were extracted and placed in a 2 mL tube containing 500 μL of Eagle’s minimum essential medium (EMEM, ICN Biomedicals, Inc.) supplemented with 20 mM HEPES and 1% penicillin-streptomycin. The tubes containing goldfish scales were placed in a ring-shaped reservoir and exposed to ELF-EMFs for 24 h. Goldfish scales were then fixed with 10% formalin in 0.05 M cacodylate buffer (pH 7.4) and stored in 0.05 M cacodylate buffer at 4°C until use.

For exposure of zebrafish to ELF-EMFs, three zebrafish were transferred to a ring-shaped tank immediately after scale cutting, and the system water was circulated through the tank via a peristaltic pump to maintain the water temperature at 28.5°C. Zebrafish were exposed to ELF-EMFs for 4 h, then transferred to the circulating aquarium, and kept for 20 h. To induce higher eddy currents in system water by ELF-EMFs without stressing the zebrafish, NaCl was added to the system water at a concentration of 0.5%. After anesthetizing with 0.02% tricaine, fractured scales were extracted from the zebrafish and fixed with 4% paraformaldehyde (PFA) in phosphate-buffered saline (PBS) overnight at 4°C. Fixed zebrafish scales were washed with a gradient of methanol in 0.1% Tween-20 (Sigma) in PBS (PBST) and kept in methanol at −20°C until use.

For ELF-EMF exposure to zebrafish fractured scales in the presence of IWR-1-endo, zebrafish with fractured stimuli were anesthetized with 0.02% tricaine, and fractured scales were extracted and placed in a 500 μL tube containing 500 μL conditioned medium (40% Leibniz’s L-15 medium (Wako), 32% Dulbecco’s modified Eagle’s medium (Wako), 12% Ham’s F12 medium (Wako), 8% FBS, 2 mM L-glutamine (Wako), 15 mM 4-(2-hydroxyethyl)-1-piperazineethanesulfonic acid (HEPES, Sigma), 100 U/mL penicillin (Wako), and 100 μg/mL streptomycin (Wako) supplemented with 20 μM of IWR-1-endo or 0.2% DMSO for control. The tubes were then placed in a ring-shaped reservoir and exposed to ELF-EMFs for 4 h. The tubes were transferred to an incubator and incubated at 28.5°C for 20 h.

### Alkaline phosphatase and tartrate-resistant acid phosphatase activity

Fixed goldfish scales were transferred to each well of a 96-well plate and alkaline phosphatase (ALP) and tartrate-resistant acid phosphatase (TRAP) activity was measured as previously described (Suzuki and Hattori, 2002). For ALP activity, an aliquot of 200 μL of alkaline buffer (100 mM Tris-HCl, pH 9.5; 100 mM NaCl; 50 mM MgCl_2_) was added to each well of a 96-well plate, followed by incubation at 20°C for 30 min with gentle agitation. After incubation, the reaction was stopped by adding 50 μL of 2 N NaOH. For TRAP activity, an aliquot of 200 μL of 10 mM para-nitrophenyl-phosphate and 20 mM tartrate in 0.1 M sodium acetate buffer (pH 5.3) was added to each well of a 96-well plate, followed by incubation at 20°C for 30 min with gentle agitation. After incubation, the reaction was stopped by adding 50 μL of 2 N NaOH. The 150 μL of colored solution was transferred to a new plate and absorbance was measured at 405 nm. The absorbance was converted to the amount of para-nitrophenol (pNP) produced using a standard curve for pNP. After measuring ALP and TRAP activities, the size of goldfish scales was measured with ImageJ. Thereafter, ALP and TRAP activities were normalized to the surface area (mm^2^) of each scale. The ALP and TRAP activity values were then shown relative to the mean value of the unexposed scale as 1.

### Flow cytometry

Cells were collected from zebrafish scales and analyzed by flow cytometry as previously described (Kobayashi-Sun et al., 2020a). Extracted scales were treated with Liberase TM (Roche) in PBS for 1 h at 37°C. The cells were then filtered through a 40 μm mesh and washed with 2% fetal bovine serum (FBS) in Hanks’ balanced salt solution (Wako) by centrifugation (300X g). Data were acquired on a FACS Aria III (BD Biosciences). Data analysis was performed using the Kaluza software (ver. 1.3, Beckman Coulter). The volume of fluid acquired per sec in FACS Area III was calculated by acquiring Accudrop beads (BD Bioscieces) whose concentrations were measured using a hemocytometer (Funakoshi). The absolute cell number was calculated based on the sample volume, acquisition events, acquisition times, and the percentage of each cell fraction.

### Immunohistochemistry and immunofluorescence

Whole-mount immunohistochemistry of zebrafish scales was performed as previously described (Kobayashi-Sun et al., 2020b). Briefly, fixed zebrafish scales were permeabilized with acetone, blocked with 2% blocking reagent (Roche), and incubated overnight at 4°C with chicken anti-GFP antibody (Aves, GFP-1020) at 1:1,000 dilution and rabbit anti-RFP antibody (for mCherry staining) (Abcam) at 1:1,000 or rabbit anti-*β*-catenin antibody (Cell Signaling Technology) at 1:100. After washing with PBST, zebrafish scales were incubated overnight at 4°C with goat anti-chicken IgY Alexa Fluor 488-conjugated (Abcam) at 1:1,000 and donkey anti-rabbit IgG Alexa Fluor 647-conjugated (Abcam) at 1:1,000 dilution. Zebrafish scales were then stained with Hoechst 33342 at 5 μg/mL in PBS for 30 min and mounted with ProLong Gold Antifade Mountant (Thermo Fisher Scientific).

Immunofluorescence of isolated cells was performed as previously described (Kimura et al., 2022). Briefly, cells were collected from zebrafish scales as described above, smeared with Cyto-tek 2500 (Sakura), and fixed with 4% PFA in PBS for 20 min at room temperature. After blocking with 0.1% gelatin-PBS, cells were stained with rabbit anti-*β*-catenin antibody at 1:100 dilution for 40 min at room temperature. After washing with 0.1% gelatin-PBS, cells were stained with donkey anti-rabbit IgG Alexa Fluor 647-conjugated (Abcam) at 1:1000 dilution for 40 min at room temperature. After washing with 0.1% gelatin-PBS, cells were stained with Hoechst 33342 and mounted with ProLong Gold Antifade Mountant as described above.

Zebrafish scales and cells were imaged using an FV10i confocal microscope and Fluoview FV10i-SW software (ver. 2.1.1) (Olympus). To compare *osterix:mCherry* and *trap:GFP* expression, the mean fluorescent intensity of mCherry per square micrometer and the percentage of coverage area of GFP were calculated in each fractured scale.

### Quantitative real-time polymerase chain reaction

Total RNA was extracted from zebrafish scales using RNeasy Mini Kit (QIAGEN) and cDNAs were synthesized with ReverTra Ace qPCR RT Master Mix (Toyobo). Quantitative real-time polymerase chain reaction (qPCR) assays were performed using TB Green Premix Ex Taq II (TaKaRa) on a ViiA 7 Real-Time PCR System according to the manufacturer’s instructions (Thermo Fisher Scientific). Expression of *ef1a* (*ef1a1l1*) was used to normalize transcript levels using the ΔΔCt method. Primer sequences used for qPCR are listed in Supplementary Table S1.

### RNA-seq and data analysis

3′RNA-seq was performed according to the Lasy-Seq Ver 1.1 method (Kamitani et al., 2019; Kashima et al., 2022). Briefly, total RNA was extracted from zebrafish scales as described above. Reverse transcription (RT) was performed using Super Script IV (Thermo Fisher Scientific) and an RT primer containing oligo-dT, the index sequence, and 9-bases unique molecular identifiers (UMIs). RNA/cDNA hybrids were purified using AMpure XP beads (Beckman Coulter), and second-strand DNA was synthesized using DNA polymerase I (Enzymatics). dsDNAs were fragmented using WGS Fragmentation Mix (Enzymatics) and ligated into customized adapters with WGS Ligase (Enzymatics). Adapter-ligated dsDNAs were amplified with KAPA HiFi ReadyMix (Nippon Genetics) and SE PCR primers. Next-generation sequencing of cDNA libraries was performed by Novogene using an Illumina NovaSeq X (illumina). Primer and adaptor sequences used for RNA-seq were listed in Supplementary Table S2.

Sequence reads were trimmed with fastp (ver. 0.21.0). The trimmed reads were then mapped to the zebrafish reference sequences generated from Danio_rerio.GRCz11 genome and version 109 gff with gffread (ver. 0.12.7), using BWA mem (version 0.7.17-r1188). Duplicated UMIs per gene in bam files were deduplicated with UMItools dedup with a parameter --per-contig. UMI counts of each transcript in each sample were calculated using salmon (ver. 1.4.0). The data have been deposited in Gene Expression Omnibus (GEO) (National Center for Biotechnology Information) and are accessible through the GEO database (series accession number, GSE247669). Gene set enrichment analysis (GSEA) was performed using the GSEA software (Broad Institute, ver. 4.1.0). Hierarchical clustering of each subset was performed in R (ver. 4.1.2) with the Bioconductor gplots package.

### Statistical analysis

Statistical significance between groups was determined by unpaired two-tailed Student’s *t*-test, one-way ANOVA with Dunnett’s test, or Pearson’s chi square test. A value of *p* < 0.05 was considered statistically significant.

## Results

### ELF-EMF exposure increases both osteoblast and osteoclast activity in the goldfish scale

**FIGURE 1:**
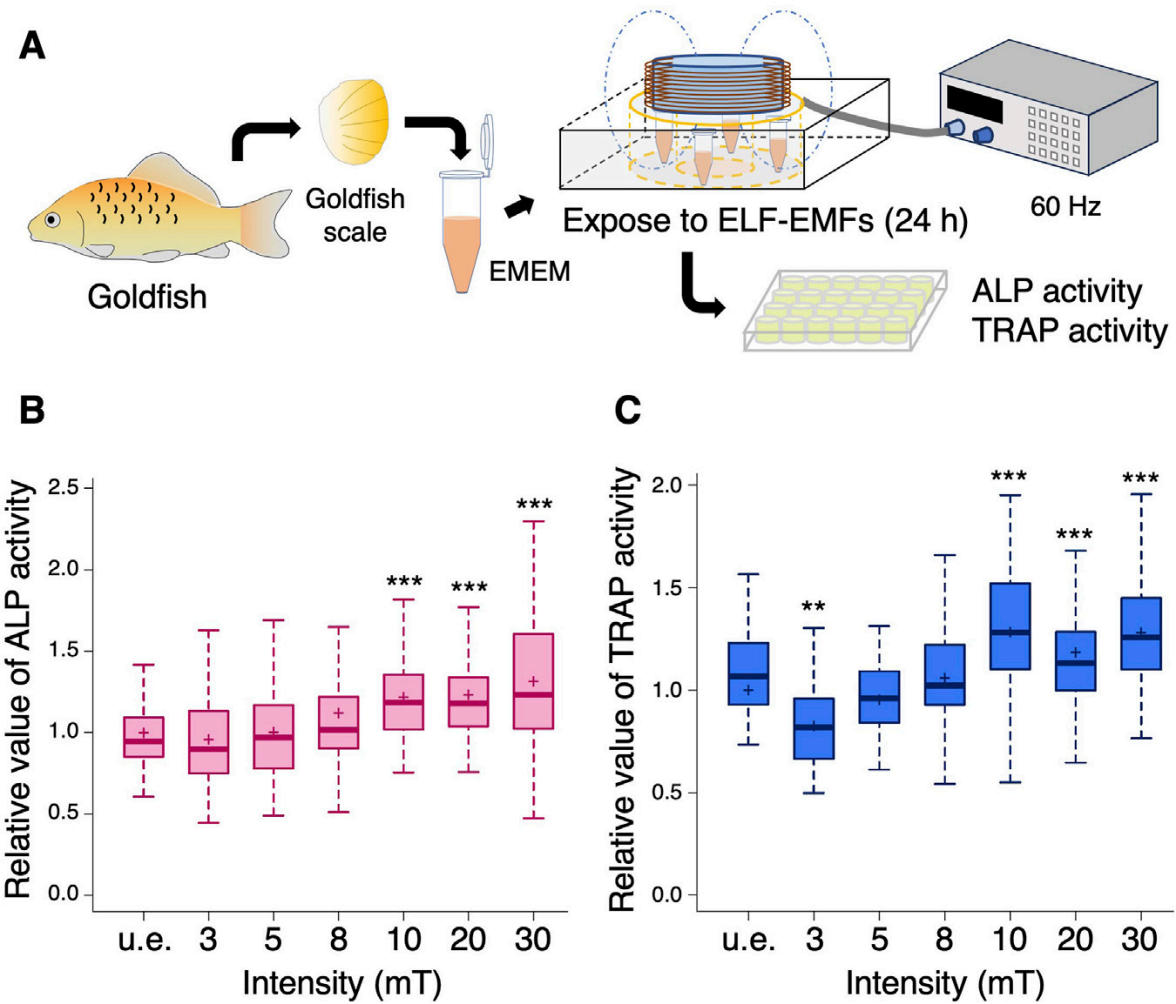
Both ALP and TRAP activity increase upon exposure to more than 10 mT of ELF-EMFs in the goldfish scale. **(A)** Schematic diagram of the experimental method. Extracted goldfish scales were unexposed (u.e.) or exposed to ELF-EMFs at varying intensities (3, 5, 8, 10, 20, or 30 mT) for 24 h, followed by measurement of ALP or TRAP activity in each goldfish scale. **(B,C)** Boxplots show the relative values of ALP **(B)** and TRAP activity **(C)** with the mean value in the u.e. scale as 1. “+” within the boxplot denotes the mean value. A total of 56–100 goldfish scales from 7 to 10 goldfish were used in each condition. Asterisks indicate the *p*-value in one-way ANOVA with Dunnett’s test vs. u.e. group. **p < 0.01, ***p < 0.001.

We first investigated the effects of ELF-EMFs at 60 Hz on ALP activity of osteoblasts and tartrate-resistant acid phosphatase (TRAP) activity of osteoclasts. Goldfish scales are larger than zebrafish ones, making it easier to test ALP and TRAP activity in the scale. Eight to ten scales per goldfish were extracted, transferred to tubes containing EMEM, and unexposed (u.e.) or exposed to ELF-EMFs at 60 Hz with varying intensities of 3, 5, 8, 10, 20, and 30 mT for 24 h (Figure 1A). Figures 1B, C show the relative values of ALP and TRAP activity at each intensity, respectively. A significant increase in both ALP and TRAP activity was observed in the goldfish scale exposed to 10, 20, and 30 mT compared to the u.e. scale, suggesting that exposure to 10 mT of ELF-EMFs may be sufficient to enhance both ALP and TRAP activity. In contrast, a significant decrease in TRAP activity was observed in the goldfish scale exposed to 3 mT compared to the u.e. scale, whereas ALP activity was unchanged.

### Exposure to 10 mT of ELF-EMFs increases both osteoblast and osteoclast activity in the zebrafish scale

**FIGURE 2:**
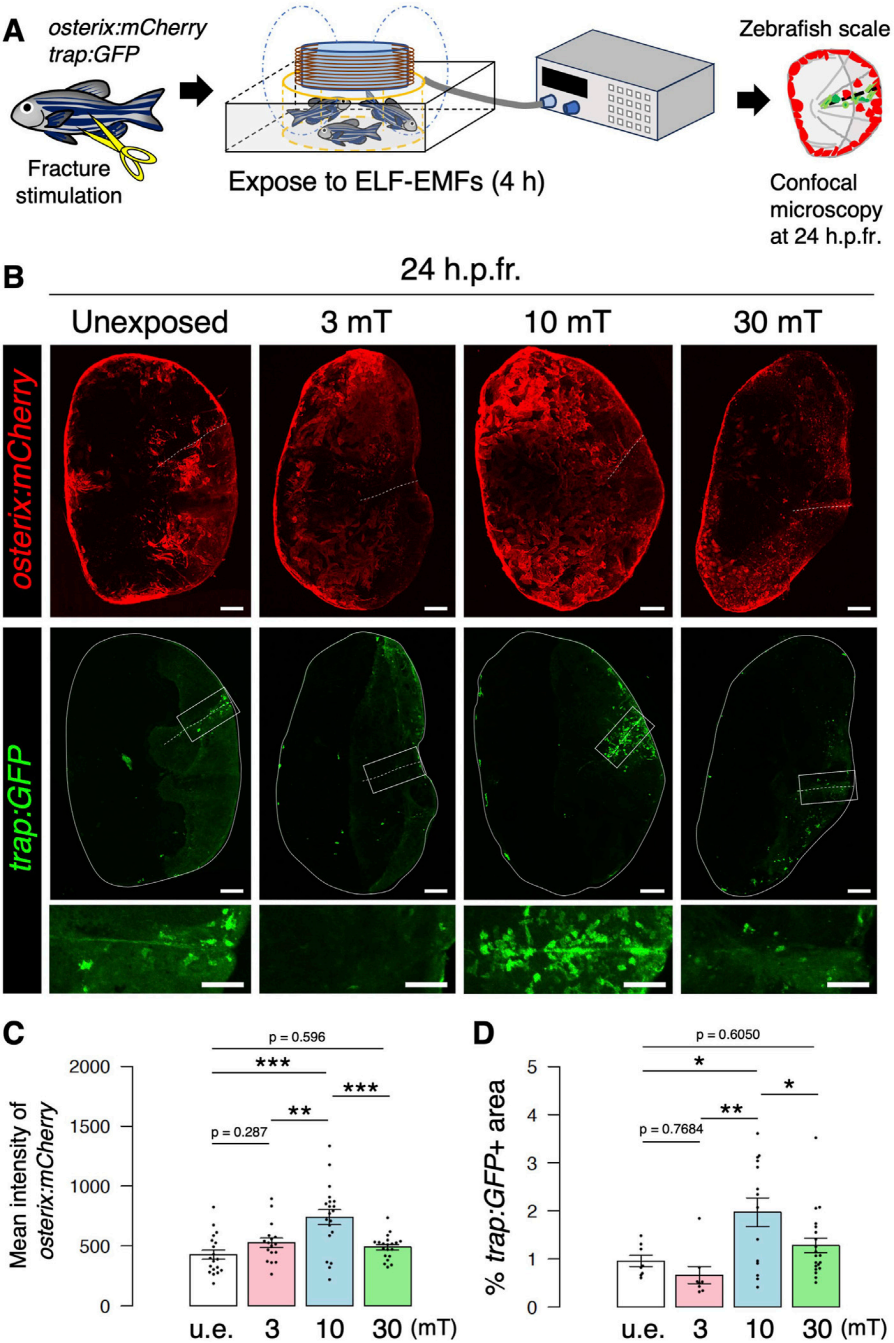
Both osteoblast and osteoclast activity increase upon exposure to 10 mT of ELF-EMFs in the zebrafish fractured scale. **(A)** Schematic diagram of the experimental method. After fracture stimulation, zebrafish were placed in a ring-shaped tank and unexposed (u.e.) or exposed to ELF-EMFs with varying intensities (3, 10, or 30 mT) for 4 h, followed by imaging of zebrafish scales by confocal microscopy. **(B)** Representative images of fractured scales in *osterix:mCherry*; *trap:GFP* double-transgenic zebrafish unexposed or exposed to ELF-EMFs at 24 h.p.fr. White dotted lines and solid lines indicate the fracture site and contour of the zebrafish scale, respectively. Lower panels of *trap:GFP* show a high magnification view of the fracture site (boxed region). Bars, 200 μm (*osterix:mCherry* and upper panels of *trap:GFP*); 100 μm (lower panels of *trap:GFP*). **(C,D)** Mean fluorescent intensity of *osterix:mCherry* per zebrafish scale and percentage of *trap:GFP* ^+^ area (coverage area) per zebrafish scale in fractured scales from zebrafish unexposed or exposed to ELF-EMFs. A total of 17–20 scales from 3 zebrafish were used in each condition. Asterisks indicate the *p*-value in one-way ANOVA with Dunnett’s test. Error bars, s.e.m.; *p < 0.05, **p < 0.01, ***p < 0.001.

To evaluate the effects of ELF-EMFs on fracture healing by osteoblasts and osteoclasts *in vivo*, we established an experimental system in which zebrafish were placed in a ring-shaped tank and exposed to ELF-EMFs while the system water was circulating at constant temperature. The epidermis portion of the scales in *trap:GFP*; *osterix:mCherry* double-transgenic zebrafish was cut with fine scissors to induce fracture, and these animals were unexposed or exposed to ELF-EMFs at 60 Hz with varying intensities of 3, 10, or 30 mT for 4 h. Zebrafish scales were then extracted at 24 h post-fracture (h.p.fr.) and analyzed by confocal microscopy (Figure 2A). Figure 2B shows representative expression patterns of *osterix:mCherry* or *trap:GFP* in the extracted scale of zebrafish unexposed or exposed to ELF-EMFs. We observed increased expression of *osterix:mCherry* throughout the fractured scale from zebrafish exposed to 10 mT (hereafter denoted as the “10 mT fractured scale”), but not in the 30 mT fractured scale. The mean fluorescent intensity of *osterix:mCherry* was approximately 1.73-fold higher in the 10 mT fractured scale compared to the u.e. fractured scale, whereas that in the 3 mT or 30 mT fractured scale was unchanged (Figure 2C), suggesting that exposure to 10 mT ELF-EMFs increases osteoblast activity. Similar results were also obtained in *trap:GFP* ^+^ osteoclasts. A large number of *trap:GFP* ^+^ osteoclasts accumulated at the fracture site in the 10 mT fractured scale, but not in the 3 or 30 mT fractured scale (Figure 2B). To compare osteoclast activity in each condition, we measured the percentage of coverage area of *trap:GFP* ^+^ within the fractured scale. The coverage area of *trap:GFP* ^+^ was approximately 2.06-fold higher in the 10 mT fractured scale than the u.e. fractured scale, but tended to be reduced in the 3 mT fractured scale, which was consistent with TRAP activity in the goldfish scale. The coverage area of *trap:GFP* ^+^ was unchanged in the 30 mT fractured scale (Figure 2D).

We followed the changes in *osterix:mCherry* and *trap:GFP* expression upon exposure to 10 mT ELF-EMFs until 3 days post-fracture (d.p.fr.). In the u.e. fractured scale, the coverage area of *trap:GFP* ^+^ increased gradually until 3 d.p.fr. In contrast, after a high increase by 2 d.p.fr., the coverage area of *trap:GFP* ^+^ began to decrease on 3 d.p.fr. in the 10 mT fractured scale, suggesting that bone resorption at the fracture site peaked out by 2 d.p.fr. in the 10 mT fractured scale. The mean fluorescent intensity of *osterix:mCherry* was unchanged by exposure to 10 mT ELF-EMFs at 2 and 3 d.p.fr. (Supplementary Figures S1A–C). These data suggest that exposure to 10 mT ELF-EMFs may have at least the effect of shortening the bone resorption period.

### Eddy currents at the tissue/cell level promote osteoblast and osteoclast activity

**FIGURE 3:**
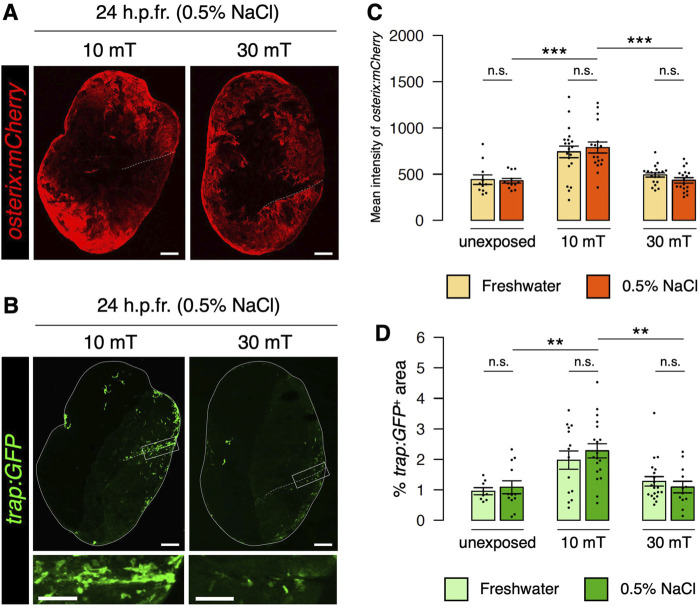
Eddy currents generated outside the body have no effect on osteoblast or osteoclast activity. **(A,B)** Representative images of fractured scales in *osterix:mCherry*; *trap:GFP* double-transgenic zebrafish exposed to 10 or 30 mT ELF-EMFs in 0.5% NaCl at 24 h.p.fr. White dotted lines and solid lines indicate the fracture site and contour of the zebrafish scale, respectively. Lower panels of *trap:GFP* show a high magnification view of the fracture site (boxed region). Bars, 200 μm (*osterix:mCherry* and upper panels of *trap:GFP*); 100 μm (lower panels of *trap:GFP*). **(C,D)** Mean fluorescent intensity of *osterix:mCherry* per scale and percentage of *trap:GFP* ^+^ area (coverage area) per zebrafish scale in fractured scales from zebrafish unexposed or exposed to 10 or 30 mT ELF-EMFs in freshwater (“Fresh”) or 0.5% NaCl (“NaCl”). The data in fresh water are used from Figures 2C,D. A total of 12–18 scales from 3 zebrafish were used in each condition. Asterisks indicate the *p*-value in one-way ANOVA with Dunnett’s test. Error bars, s.e.m.; **p < 0.01, ***p < 0.001, n.s., not significant.

Since ELF-EMF exposure generates eddy currents strongly in saline water but weakly in freshwater, it may be possible that the effects of ELF-EMFs on the goldfish scale in EMEM and those on the zebrafish scale in freshwater are distinct. Therefore, we investigated the expression of *osterix:mCherry* and *trap:GFP* under conditions in which zebrafish are unexposed or exposed to 10 or 30 mT ELF-EMFs in system water containing 0.5% NaCl. As in freshwater, higher expression of *osterix:mCherry* and a greater number of *trap:GFP* ^+^ osteoclasts was observed in the 10 mT fractured scale in 0.5% NaCl compared to the unexposed or 30 mT fractured scale in 0.5% NaCl (Figures 3A, B). The mean fluorescent intensity of *osterix:mCherry* and the coverage area of *trap:GFP* ^+^ was approximately 1.82-fold and 2.09-fold higher in the 10 mT fractured scale compared to the 30 mT fractured scale in 0.5% NaCl, respectively, which were similar to those in freshwater (1.51-fold and 1.54-fold, respectively). We observed no significant differences in either the mean fluorescent intensity of *osterix:mCherry* or the coverage area of *trap:GFP* ^+^ between freshwater and 0.5% NaCl at 10 or 30 mT as well as unexposed controls (Figures 3C, D). These results suggest that eddy currents generated within the tissues/cells of the zebrafish scale, but not outside the body, promote the activity of both osteoblasts and osteoclasts.

### Exposure to 10 mT of ELF-EMFs increases both osteoblast and osteoclast numbers

**FIGURE 4:**
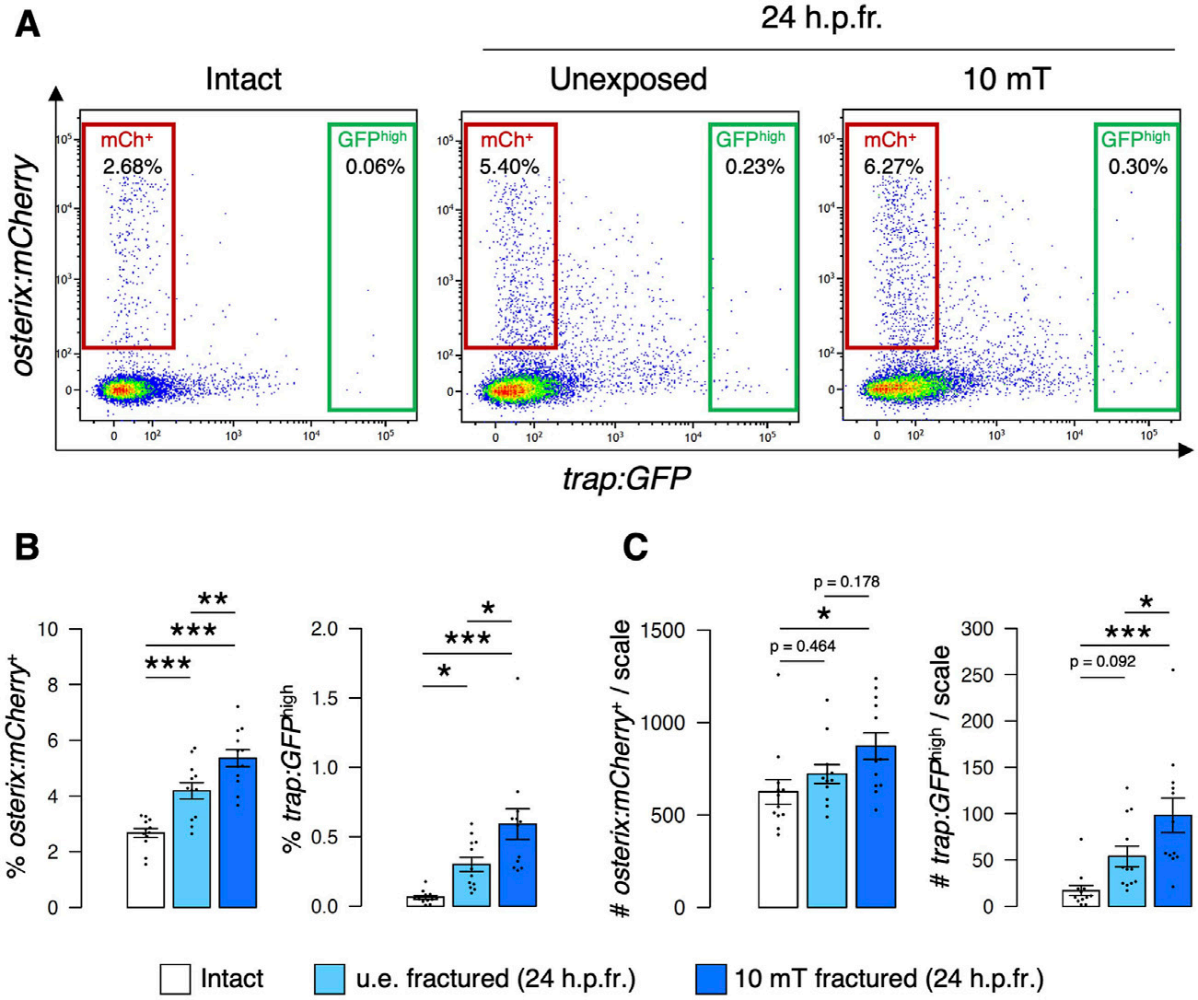
Both osteoblast and osteoclast numbers increase upon exposure to 10 mT of ELF-EMFs in the fractured scale. **(A)** Representative results of flow cytometric analysis of cells from intact scales (left panel), unexposed (u.e.) fractured scales (middle panel), or fractured scales exposed to 10 mT ELF-EMFs (right panel) at 24 h.p.fr. After fracture stimulation, zebrafish were placed in the ring-shaped tank and were unexposed or exposed to 10 mT ELF-EMFs for 4 h. Zebrafish scales were extracted at 24 h.p.fr., and cell samples were prepared for flow cytometric analysis. Red and green gates show *trap:GFP* ^–^ *osterix:mCherry* ^+^ (“mCh^+^”) and *trap:GFP* ^high^ (“GFP^high^”) cells, respectively. **(B,C)** Percentage **(B)** and absolute number of mCh^+^ and GFP^high^ cells per scale **(C)** in intact scales, unexposed fractured scales (u.e. fractured), or fractured scales exposed to 10 mT ELF-EMFs (10 mT fractured). A total of 12 samples from 6 zebrafish were used in each condition. Asterisks indicate the *p*-value in one-way ANOVA with Dunnett’s test. Error bars, s.e.m.; *p < 0.05, **p < 0.01, ***p < 0.001.

To further investigate the effect of ELF-EMFs on osteoblasts and osteoclasts, we next quantified the number of osteoblasts and osteoclasts by flow cytometry. We have previously shown that mature osteoclasts with one to three nuclei are detected in the *trap:GFP* ^high^ fraction at 24 h.p.fr., whereas immature osteoclasts are in the *trap:GFP* ^low^ fraction. Due to uptake of osteoblast-derived extracellular vesicles, the majority of *trap:GFP* ^+^ osteoclasts are detected in the *osterix:mCherry* ^+^ fraction (Kobayashi-Sun et al., 2020a) (Supplementary Figure S2). The percentage of both *osterix:mCherry* ^+^ osteoblasts (“mCh^+^”) and *trap:GFP* ^high^ osteoclasts (“GFP^high^”) was increased in the u.e. fractured scale compared to the intact scale, as shown in our previous study (Kobayashi-Sun et al., 2020a). These percentages were further raised in the 10 mT fractured scale under freshwater conditions (Figures 4A, B). We also quantified the absolute numbers of *osterix:mCherry* ^+^ osteoblasts and *trap:GFP* ^high^ osteoclasts per zebrafish scale in each condition. Despite an increase in the percentage of *osterix:mCherry* ^+^ osteoblasts with fracture stimulation, there was no significant change in the number of osteoblasts between the u.e. fractured scale and the intact scale. This is attributed to the decreased number of total cells in the fractured scale due to scale cutting. However, there was a significant increase in the number of *osterix:mCherry* ^+^ osteoblasts in the 10 mT fractured scale compared to the intact scale. There was also a significant increase in the number of *trap:GFP* ^high^ osteoclasts in the 10 mT fractured scale compared to the intact scale or the u.e. fractured scale (Figure 4C). These data suggest that exposure to 10 mT ELF-EMFs leads to increase in the number of both osteoblasts and osteoclasts in the fractured scale.

The percentage and number of *osterix:mCherry* ^+^ osteoblasts and *trap:GFP* ^high^ osteoclasts were also examined in the intact scale unexposed or exposed to 10 mT ELF-EMFs. An increase in the percentage and number of osteoclasts was observed in 10 mT intact scales compared to u.e. intact scales, while the increase in osteoblasts was not significant (Supplementary Figures S3A–C).

### Wnt and Notch signaling are enhanced by ELF-EMF exposure

**FIGURE 5:**
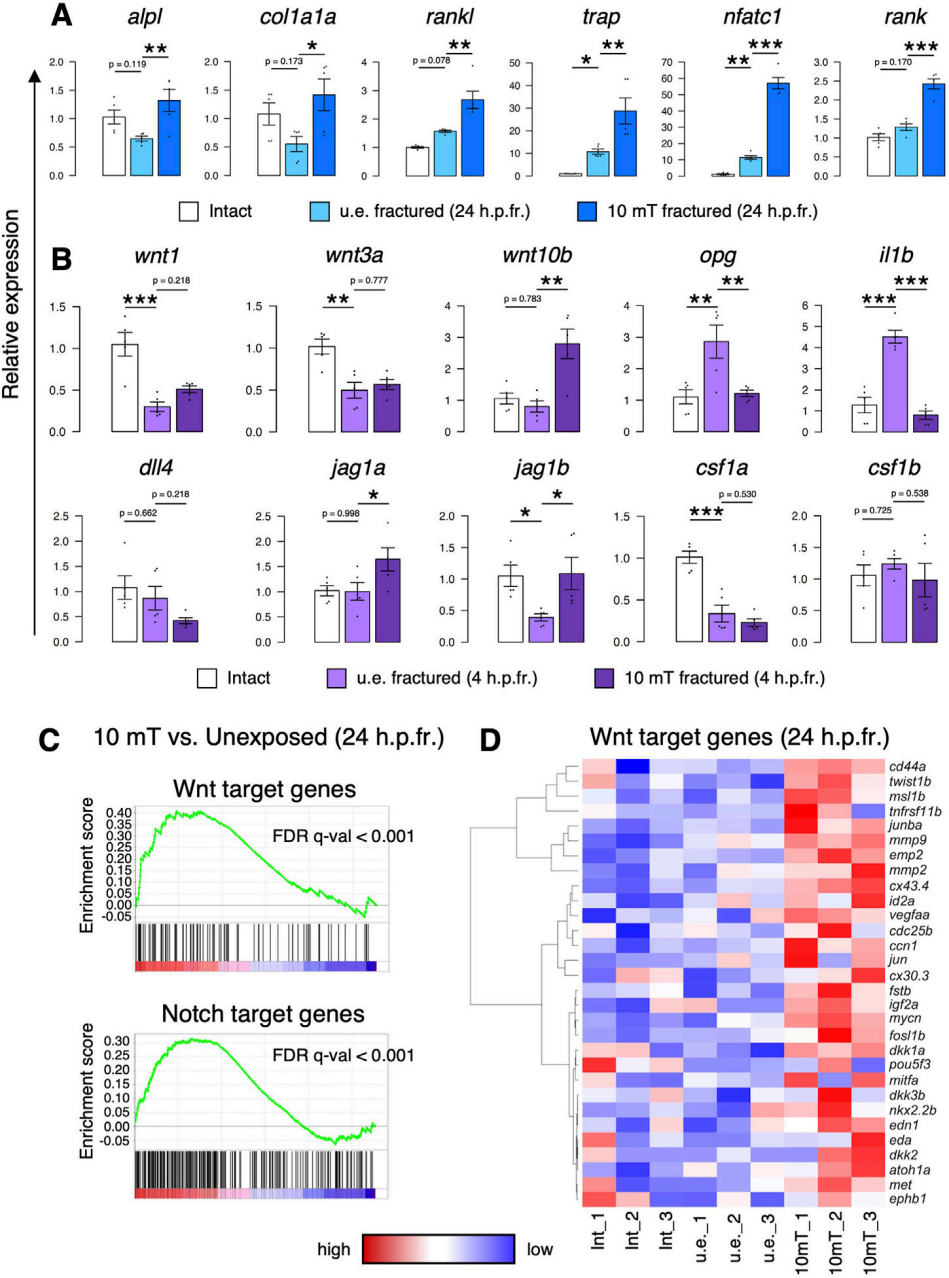
Exposure to 10 mT of ELF-EMFs enhances Wnt and Notch signaling in the fractured scale. **(A,B)** Relative gene expression of osteoblast markers (*alpl*, *col1a1a*, and *rankl*) and osteoclast markers (*trap*, *nfatc*, and *rank*) in intact scales, unexposed fractured scales (u.e. fractured), or fractured scales exposed 10 mT ELF-EMFs (10 mT fractured) at 24 h.p.fr. **(A)** and signaling molecules for osteoblasts and/or osteoclasts in intact scales, u.e. fractured scales, or 10 mT fractured scales at 4 h.p.fr. **(B)**. Relative expression levels were calculated by ΔΔCt method with the reference gene of *ef1a*. Asterisks indicate the *p*-value in one-way ANOVA with Dunnett’s test. Error bars, s.e.m. (n = 5 for each). *p < 0.05, **p < 0.01, ***p < 0.001. **(C)** Gene set enrichment analysis (GSEA) of Wnt target genes (upper panel) and Notch target genes (lower panel) in fractured scales unexposed or exposed to 10 mT ELF-EMFs at 24 h.p.fr. (*n* = 3 for each) **(D)** Hierarchical clustering of selected Wnt target genes in intact scales (Int_1–3), u.e. fractured scales (u.e._1–3), or 10 mT fractured scales (10 mT_1–3) at 24 h.p.fr. (*n* = 3 for each).

To investigate the molecular mechanisms underlying the effect of 10 mT ELF-EMFs on osteoblasts and osteoclasts, gene expression analysis was performed in the zebrafish scale. After fracture stimulation, zebrafish were unexposed or exposed to 10 mT ELF-EMFs for 4 h, followed by extraction of zebrafish scales at 24 h.p.fr. for qPCR analysis. As shown in Figure 5A, the expression of both osteoblast marker genes (*alpl*, *col1a1a*, and *rankl*) and osteoclast marker genes (*trap*, *nfatc1*, and *rank*) increased in the 10 mT fractured scale compared to the u.e. fractured scale or the intact scale. Since the number of osteoblasts and osteoclasts was increased by exposure to 10 mT ELF-EMFs, it is likely that proliferation signals for osteoblasts and/or osteoclasts may be increased in the 10 mT fractured scale. Therefore, fractured scales were extracted from zebrafish immediately after exposure to 10 mT ELF-EMFs and examined the mRNA expression of some critical signaling molecules at 4 h.p.fr. We found that the expression of canonical *wnt* genes, *wnt10b*, and Notch ligand genes, such as *jag1a* and *jag1b*, increased in the 10 mT fractured scale compared to the u.e. fractured scale. Particularly, *wnt10b* was expressed approximately 3.6-fold higher in the 10 mT fractured scale than in the u.e. fractured scale. In contrast, the expression of *opg* and *il1b* is upregulated by fracture stimulation but is reduced by exposure to 10 mT ELF-EMFs (Figure 5B). To examine whether Wnt and/or Notch signaling is enhanced by ELF-EMF exposure, RNA-seq analysis was performed on scales from zebrafish unexposed or exposed to 10 mT ELF-EMFs at 24 h.p.fr. Gene set enrichment analysis (GSEA) revealed that both Wnt target genes and Notch target genes significantly increased in the 10 mT fractured scale compared to the u.e. fractured scale (Figure 5C). Since the *β*-catenin-dependent canonical Wnt pathway has been shown to promote proliferation and differentiation of osteoblast precursors (Kato et al., 2002; Clément-Lacroixet al., 2005), the increased numbers of osteoblasts and osteoclasts by ELF-EMF exposure may be a consequence of enhanced canonical Wnt signaling. Indeed, some Wnt target genes involved in cell proliferation, such as *mycn*, *jun*, *igf2a*, *edn1*, and *cd44a*, were highly expressed in the 10 mT fractured scale compared to the u.e. fractured scale (Figure 5D).

### Wnt/*β*-catenin signaling is induced by ELF-EMF exposure at the fracture site

**FIGURE 6:**
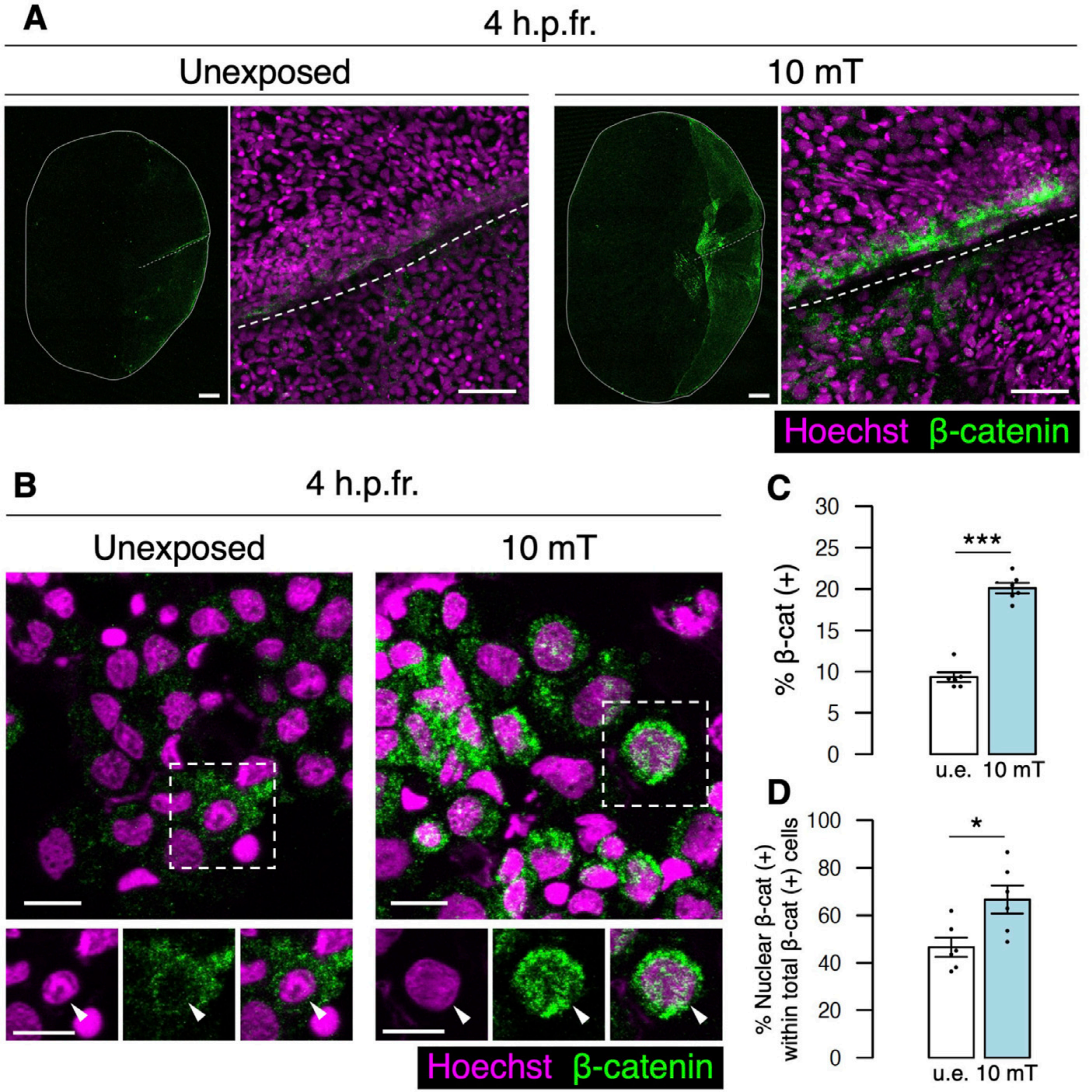
Expression of *β*-catenin is increased at the fracture site upon exposure to 10 mT of ELF-EMFs. **(A)** Expression of *β*-catenin in the fractured scale unexposed (u.e.) or exposed to 10 mT ELF-EMFs at 4 h.p.fr. After fracture stimulation, zebrafish were placed in the ring-shaped tank and were unexposed or exposed to 10 mT ELF-EMFs for 4 h. Zebrafish scales were then stained with rabbit anti-β-catenin antibody, followed by anti-rabbit IgG Alexa Fluor 647-conjugated secondary antibody and Hoechst 33342. White dotted lines and solid lines indicate the fracture site and contour of the zebrafish scale, respectively. Right panels show a high magnification view of the fracture site. Bars, 200 μm (left panels) and 40 μm (right panels). **(B)** Expression of *β*-catenin in cells from fractured scales unexposed or exposed to 10 mT ELF-EMFs at 4 h.p.fr. After fracture stimulation, zebrafish were placed in the ring-shaped tank and were unexposed or exposed to 10 mT ELF-EMFs for 4 h. Cells were then collected from zebrafish scales, smeared, and stained with rabbit anti-β-catenin antibody, followed by anti-rabbit IgG Alexa Fluor 647-conjugated secondary antibody and Hoechst 33342. Bottom panels show Hoechst (nuclei), *β*-catenin expression, and merged images of the dotted region. Arrowheads indicate the nucleus. Bars, 10 μm. **(C,D)** Percentage of *β*-catenin (+) cells within total cells and percentage of nuclear *β*-catenin (+) cells within total *β*-catenin (+) cells in the u.e. fractured scale or the fractured scale exposed to 10 mT ELF-EMFs at 4 h.p.fr. A total of 6 samples from 3 zebrafish were used in each condition. Asterisks indicate the *p*-value in unpaired two-tailed Student’s t-test. Error bars, s.e.m.; *p < 0.05.; ***p < 0.001.

Canonical Wnt signaling stabilizes *β*-catenin by preventing *β*-catenin phosphorylation from the destruction complex, which results in translocation of *β*-catenin into the nucleus to transcribe Wnt target genes (MacDonald et al., 2009; Liu et al., 2022). To examine whether *β*-catenin expression is enhanced by exposure to 10 mT ELF-EMFs, zebrafish with fracture stimulation were unexposed or exposed to 10 mT ELF-EMFs for 4 h, and fractured scales were extracted and stained with anti-*β*-catenin antibody. The expression of *β*-catenin was strongly detected in the 10 mT fractured scale, whereas it was very weak in the u.e. fractured scale. Interestingly, *β*-catenin expression signals were predominantly observed along the fractured site within the zebrafish scale (Figure 6A), suggesting that Wnt signaling is induced at the fracture site by ELF-EMF exposure. To determine the percentage of *β*-catenin (+) cells and the localization of *β*-catenin within the cell, cells collected from the zebrafish scale were smeared and stained with anti-*β*-catenin antibody. The expression of *β*-catenin was frequently detected not only in the cytoplasm but also in the nucleus in cells from the 10 mT fractured scale (Figure 6B). The percentage of *β*-catenin (+) cells was approximately 20.1% in the 10 mT fractured scale, whereas it was approximately 9.3% in the u.e. fractured scale (Figure 6C). Moreover, the percentage of nuclear *β*-catenin (+) cells out of total *β*-catenin (+) cells was approximately 66.7% in the 10 mT fractured scale, which was significantly higher than the u.e. fractured scale (46.5%) (Figure 6D). We also examined *β*-catenin expression in *osterix:mCherry* ^+^ osteoblasts and *trap:GFP* ^+^ osteoclasts. The percentage of *osterix:mCherry* ^+^ osteoblasts within total *β*-catenin (+) cells was approximately 6.6% ± 2.2% and 7.2% ± 5.1% (±s.e.m.; *n* = 6) in the u.e. fractured and 10 mT fractured scale, respectively, whereas that of *trap:GFP* ^+^ osteoclasts was less than 0.1% in both types of the fractured scale. The percentage of nuclear *β*-catenin (+) osteoblasts out of total osteoblasts was approximately 48.5% in the 10 mT fractured scale, significantly higher than in the u.e. fractured scale (17.3%). In contrast, there was no significant difference in the percentage of nuclear *β*-catenin (+) in osteoclasts (Supplementary Figures S4A, B). Although more than 90% of *β*-catenin (+) cells were not osteoblasts or osteoclasts, elevated expression of *β*-catenin in osteoblasts was observed in the 10 mT fractured scale. Taken together, these data suggest that exposure to 10 mT ELF-EMFs enhances Wnt/β-catenin signaling particularly at the fracture site of the zebrafish scale.

### Inhibition of Wnt/*β*-catenin signaling reduces both osteoblasts and osteoclasts

**FIGURE 7:**
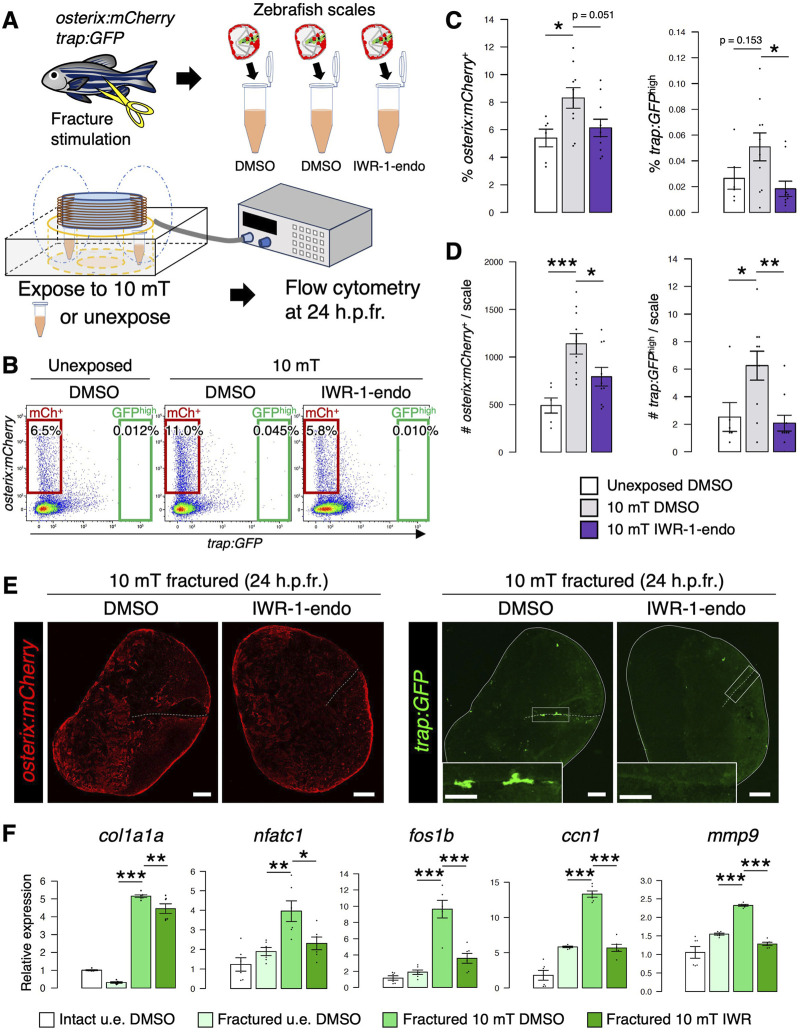
Inhibition of Wnt/*β*-catenin signaling reduces both osteoblasts and osteoclasts in the ELF-EMF exposed scale. **(A)** Schematic diagram of the experimental method. After fracture stimulation, zebrafish scales were extracted, placed in a tube containing conditioned medium supplemented with DMSO or IWR-1-endo (20 μM), and unexposed or exposed to 10 mT ELF-EMFs for 4 h. Cells were then collected from the zebrafish scales at 24 h.p.fr. and analyzed by flow cytometry. **(B)** Representative results of flow cytometric analysis of cells from fractured scales unexposed or exposed to 10 mT ELF-EMFs in the presence of DMSO or IWR-1-endo at 24 h.p.fr. Red and green gates show *trap:GFP* ^–^ *osterix:mCherry* ^+^ (“mCh^+^”) and *trap:GFP* ^high^ (“GFP^high^”) cells, respectively. **(C,D)** Percentage **(C)** and absolute number of mCh^+^ and GFP^high^ cells per zebrafish scale **(D)** in fractured scales unexposed or exposed to 10 mT ELF-EMFs in the presence of DMSO or IWR-1-endo. A total of 10 samples from 5 zebrafish were used in each condition. Asterisks indicate the *p*-value in one-way ANOVA with Dunnett’s test. Error bars, s.e.m. **(E)** Representative images of fractured scales in *osterix:mCherry*; *trap:GFP* double-transgenic zebrafish exposed to 10 mT ELF-EMFs in the presence of DMSO or IWR-1-endo at 24 h.p.fr. White dotted lines and solid lines indicate the fracture site and contour of the zebrafish scale, respectively. Insets of *trap:GFP* show a high magnification view of the fracture site (boxed region). Bars, 200 μm (*osterix:mCherry* and *trap:GFP*); 100 μm (insets of *trap:GFP*). **(F)** Relative gene expression of an osteoblast marker (*col1a1a*), osteoclast marker (*nfatc*), and Wnt target genes (*fos1b*, *ccn1*, and *mmp9*) in the intact DMSO-treated, unexposed fractured DMSO-treated, 10 mT fractured DMSO-treated, and 10 mT fractured IWR-1-endo-treated scale at 24 h.p.fr. Asterisks indicate the *p*-value in one-way ANOVA with Dunnett’s test. Error bars, s.e.m. (*n* = 6 for each). *p < 0.05, **p < 0.01, ***p < 0.001.

To test whether Wnt/*β*-catenin signaling promotes the proliferation of osteoblasts and osteoclasts in the fractured scale, we inhibited this signaling by treatment with IWR-1-endo, which promotes *β*-catenin phosphorylation via stabilization of the Axin-scaffold destruction complex (Huang et al., 2009). After fracture stimulation, zebrafish scales were extracted and transferred to conditioned medium supplemented with dimethyl sulfoxide (DMSO) or IWR-1-endo and exposed to 10 mT ELF-EMFs for 4 h. Cells were then collected from fractured scales at 24 h.p.fr. and analyzed by flow cytometry (Figure 7A). The number of both *osterix:mCherry* ^+^ osteoblasts and *trap:GFP* ^high^ osteoclasts was significantly increased by exposure to 10 mT ELF-EMFs, but it was decreased by IWR-1-endo treatment. Similar results were also obtained in the percentage of each cell type (Figures 7B–E). We also performed gene expression analysis of an osteoblast marker gene (*col1a1a*), an osteoclast marker gene (*nfatc1*), and three Wnt target genes (*fos1b*, *ccn1*, and *mmp9*) in four different types of scales, intact DMSO-treated, unexposed fractured DMSO-treated, 10 mT fractured DMSO-treated, and 10 mT fractured IWR-1-endo-treated. All of these five genes showed similar expression patterns; they increased by exposure to 10 mT ELF-EMFs, but decreased by IWR-1-endo treatment (Figure 7F). Taken together, these data suggest that Wnt/*β*-catenin signaling enhanced by ELF-EMF exposure promotes proliferation of both osteoblasts and osteoclasts under fracture stress.

## Discussion

In the present study, we demonstrated in zebrafish that exposure to 10 mT of ELF-EMFs at 60 Hz promotes both osteoblast and osteoclast activity through activation of Wnt/β-catenin signaling in the fractured scale. These results provide *in vivo* evidence that ELF-EMFs have a facilitative effect on fracture healing.

There is a close relationship between the frequency of EMFs and their effects on cells. Liu et al. showed the effects of 1 mT sinusoidal EMFs of varying frequencies on rat bone marrow mesenchymal stem cells. Exposure to 10 or 50 Hz EMFs for 7 days increased cell numbers, whereas exposure to 70 Hz EMFs conversely reduced viability by 40%. Moreover, exposure to 50 Hz EMFs increased the mRNA expression of *osteocalcin* (an osteoblast marker), *BMP2* (an osteogenic growth factor), and *ALP*, suggesting that 50 Hz EMFs may have a stimulatory effect on osteoblast proliferation (Liu et al., 2013). We examined the effects of ELF-EMFs at 60 Hz on the fish scale. Exposure to 10 mT of ELF-EMFs leads to increase both ALP and TRAP activity in the goldfish scale. Moreover, it also increased the number of osteoblasts and osteoclasts in zebrafish fractured scales. These results suggest that ELF-EMFs at 60 Hz also have a stimulatory effect on both osteoblasts and osteoclasts. Since the frequency of 50–60 Hz is the most widely used in commercial AC power supply, ELF-EMFs at 50–60 Hz are promising medical devices for the treatment of bone diseases.

The molecular mechanisms of osteoblast or osteoclast differentiation have been investigated mostly by *in vitro* cell culture experiments in mammals (Stein and Lian, 1993; Hurley et al., 1998; Rissanen et al., 2008; Shao et al., 2018; Madel et al., 2020; Liu et al., 2023a; Liu et al., 2023b). Similarly, the effects of EMFs on osteoblasts or osteoclasts have mostly been shown *in vitro* (Liu et al., 2013; Pi et al., 2019; Zhou et al., 2019), leaving open the possibility of different effects *in vitro* and *in vivo* due to eddy currents that are generated in a conductor, such as saline water, by changing the magnetic field (Yamaguchi et al., 2006). Soda et al. showed that cellular functions are affected by exposure to sinusoidal EMFs, but not by exposure to static EMFs, which do not generate eddy currents (Soda et al., 2008), suggesting that the effect of the magnetic field is mainly due to eddy currents rather than the magnetic field itself. Since eddy currents increase with the thickness of the conductor (Razavi et al., 2014), eddy currents in the culture medium may be stronger than those in the zebrafish scale. Taking advantage of the zebrafish model, we attempted to generate eddy currents outside the body/tissue by exposing zebrafish to ELF-EMFs in 0.5% NaCl. Eddy currents induced by sinusoidal EMFs are proportional to the liquid conductivity and EMF intensity. The conductivities of system water, 0.5% NaCl, and saline (0.9% NaCl) are estimated to be approximately 0.01, 0.92, and 1.60, respectively. These indicate that eddy currents in 0.5% NaCl are approximately 58% of those in saline but 92 times greater than those in system water. Moreover, eddy currents at 30 mT in 0.5% NaCl are approximately 1.67 times higher than those at 10 mT in saline. Although zebrafish scales are distributed on the body surface and much higher eddy currents are induced in 0.5% NaCl than in system water, no significant differences in either *osterix:mCherry* or *trap:GFP* expression were observed between freshwater and 0.5% NaCl after exposure to 10 mT or 30 mT ELF-EMFs. These results suggest that the promoting effect of 10 mT ELF-EMFs on osteoblasts and osteoclasts is induced by eddy currents at the tissue and/or cellular level, but not by eddy currents generated outside the body or in the culture medium.

The Wnt/*β*-catenin pathway is highly conserved and plays critical roles in regulating bone homeostasis (Baron and Kneissel, 2013). One mechanism whereby the canonical Wnt pathway mediates bone mass is believed to be the stimulation of osteoblastogenesis (Matsuzaki et al., 2013). Wnt10b has been implicated in enhancing osteoblast differentiation through the canonical Wnt signaling pathway (Bennett et al., 2005; Bennett et al., 2007; Karner and Long, 2017). A recent *in vitro* study using isolated osteoblasts showed that *Wnt10b* is upregulated by sinusoidal EMFs and promotes osteoblast differentiation (Zhou et al., 2019). Glass et al. previously reported a high bone mass phenotype in mice expressing a dominant active form of *β*-catenin in osteoblasts. They found that Wnt/*β*-catenin signaling in osteoblasts induced the expression of Osteoprotegerin (Opg), which is an inhibitory factor for osteoclast differentiation (Glass et al., 2005). Non-canonical Wnt ligands, such as Wnt16 and Wnt4, inhibit osteoclast differentiation, whereas Wnt5a enhances osteoclast formation (Kobayashi et al., 2015; Uehara et al., 2018; Maeda et al., 2019). However, whether Wnt signaling induced by EMFs actually contributes to osteoblast proliferation in bone tissue has not been fully investigated. In addition, the effect of enhanced Wnt/*β*-catenin signaling on osteoclasts also remained elusive. In the present study, we demonstrated that exposure to 10 mT of ELF-EMFs leads to increased expression of *wnt10b* and *β*-catenin but decreased expression of *opg* in the fractured scale. Inhibition of Wnt/*β*-catenin signaling by IWR-1-endo treatment resulted in reduced numbers of both osteoblasts and osteoclasts in the fractured scale exposed to ELF-EMFs. Although the molecular mechanisms by which *opg* expression remains low despite elevated Wnt signaling upon 10 mT exposure are still elusive, our data provided *in vivo* evidence that exposure to ELF-EMFs increases both osteoblasts and osteoclasts through activation of Wnt/β-catenin signaling in bone tissue.

Previous studies in mammalian cells reported that PEMFs have a promoting effect on osteoblasts, but an inhibitory effect on osteoclasts (Esposito et al., 2012; Zhou et al., 2013; Pi et al., 2019; Di Bartolomeo et al., 2022). However, the effects of PEMFs on osteoclasts still remained unsettled, and conflicting results have been reported (Shankar et al., 1998; Chang et al., 2003; Chang et al., 2005; Barnaba et al., 2012). Shankar et al. reported that PEMFs promoted bone resorption only when osteoclasts were co-cultured with osteoblasts, whereas PEMFs had no effect on bone resorption when osteoclasts were cultured alone, suggesting that osteoblasts mediate resorptive effects of PEMFs (Shankar et al., 1998). Our data in the goldfish scale showed that exposure to 10 mT of ELF-EMFs has a promoting effect on both osteoblasts and osteoclasts. In contrast, exposure to 3 mT of ELF-EMFs reduces osteoclast activity, while it had no effect on osteoblast activity. These data suggest that although the direct action of EMFs on osteoclasts may be inhibitory, osteoblasts activated by EMFs likely activate osteoclast differentiation in bone tissue, which in turn have a facilitative effect on resorptive activity. This facilitative effect of bone formation and resorption is also dependent on the timing and period of exposure; a short period of ELF-EMF exposure at an early timing from fracture stimulation may be effective for both osteoblasts and osteoclasts.

The fish scale is a thin membranous bone embedded in the skin, composed of bone matrix and many cell types, including osteoblasts, osteoclasts, epithelial cells, endothelial cells, mesenchymal cells, and multitype of immune cells. Regeneration and repair of fish scales occurs based on intramembranous ossification, indicating that the process of bone repair is slightly different between fish scales and mammalian bones, which are mostly repaired by endochondral ossification (Bergen et al., 2019; Dietrich et al., 2021). However, various *in vivo* and *in vitro* studies have demonstrated that osteoblasts and osteoclasts in fish scales respond to hormonal and molecular signals in the same manner as in mammals, indicating that fundamental cellular and molecular programs that regulate osteoclasts and osteoblasts are highly conserved between fish scales and mammalian bones (Pasqualetti et al., 2012; Aman et al., 2018; Hirayama et al., 2023). In addition, the zebrafish scale offers significant advantages for live imaging and pharmacological screening, making it a useful model to study osteoblast and osteoclast interactions and their regulatory mechanisms. Taking advantage of the zebrafish scale, further studies will provide *in vivo* evidence on the molecular mechanisms involved in osteoblast and osteoclast regulation upon exposure to ELF-EMFs.

## Data Availability

The datasets presented in this study can be found in online repositories. The names of the repository/repositories and accession number(s) can be found below: https://www.ncbi.nlm.nih.gov/, GSE247669.
