# Supplementary material for: Extremely low-frequency electromagnetic fields facilitate both osteoblast and osteoclast activity through Wnt/β-catenin signaling in the zebrafish scale
Source: Front Cell Dev Biol. 2024 Feb 7;12:1340089. doi: 10.3389/fcell.2024.1340089 (PMC10879286; doi:10.3389/fcell.2024.1340089)
Supplement: Supplementary file 1 [file DataSheet1.PDF]

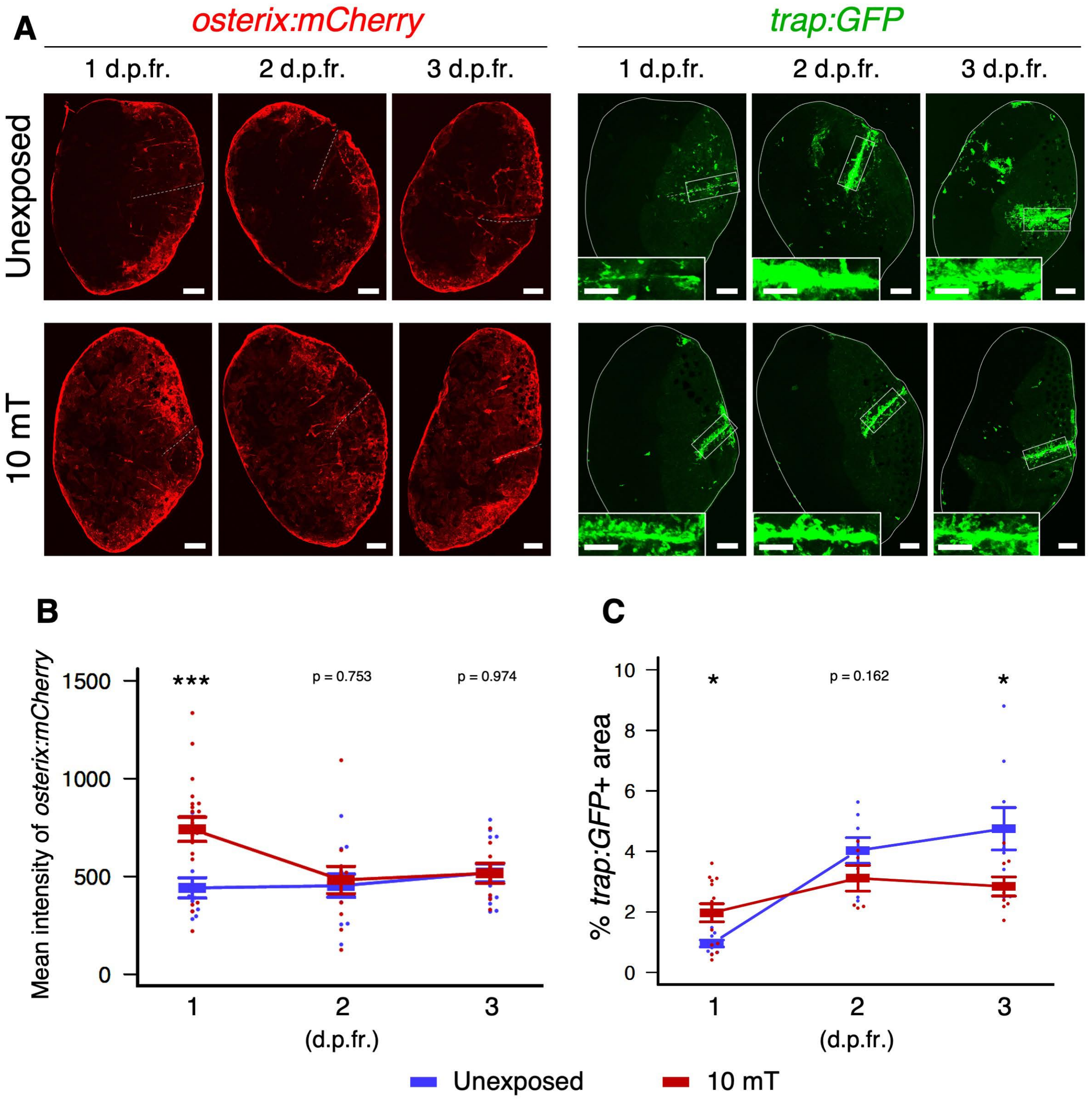

Supplementary Figure 1. Time course changes in *osterix:mCherry* and *trap:GFP* expression in the fractured scale.

(**A**) Representative images of fractured scales in *osterix:mCherry*; *trap:GFP* double-transgenic zebrafish unexposed or exposed to ELF-EMFs at 1, 2, and 3 days post-fracture (d.p.fr.). After fracture stimulation, zebrafish were placed in a ring-shaped tank and unexposed or exposed to 10 mT ELF-EMFs for 4 hours, followed by imaging of zebrafish scales by confocal microscopy at each time point. White dotted lines and solid lines indicate the fracture site and contour of the zebrafish scale, respectively. Insets of *trap:GFP* show a high magnification view of the fracture site (boxed region). Bars, 200 µm (*osterix:mCherry* and *trap:GFP*); 100 µm (insets of *trap:GFP*). (**B**, **C**) Mean fluorescent intensity of *osterix:mCherry* per zebrafish scale and percentage of *trap:GFP*$^{+}$ area (coverage area) per zebrafish scale in fractured scales from zebrafish unexposed or exposed to 10 mT ELF-EMFs. The data at 1 d.p.fr. are used from Figure 2C and D. A total of 6-10 scales from 3 zebrafish were used in each condition. *$p < 0.05$; ***$p < 0.001$.

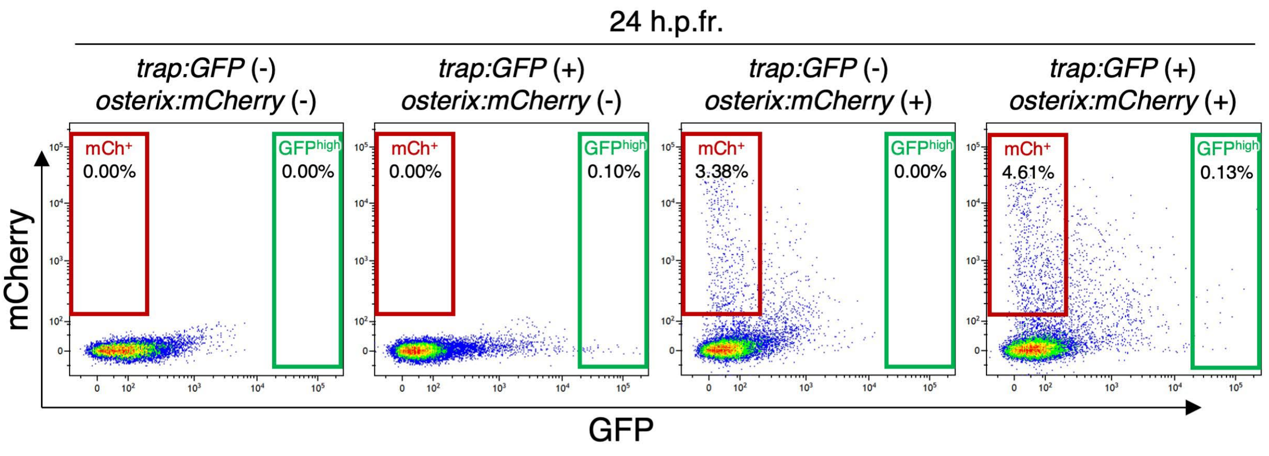

Supplementary Figure 2. Fluorescence controls of *osterix:mCherry* and *trap:GFP* expression in the fractured scale.

Representative results of flow cytometric analysis of cells in the fractured scale (24 h.p.fr.) from four different zebrafish, *trap:GFP* (-) *osterix:mCherry* (-), *trap:GFP* (+) *osterix:mCherry* (-), *trap:GFP* (-) *osterix:mCherry* (+), and *trap:GFP* (+) *osterix:mCherry* (+). Red and green gates show *trap:GFP*$^{-}$ *osterix:mCherry*$^{+}$ ("mCh^{+}") and *trap:GFP*^{high} ("GFP^{high}") cells, respectively.

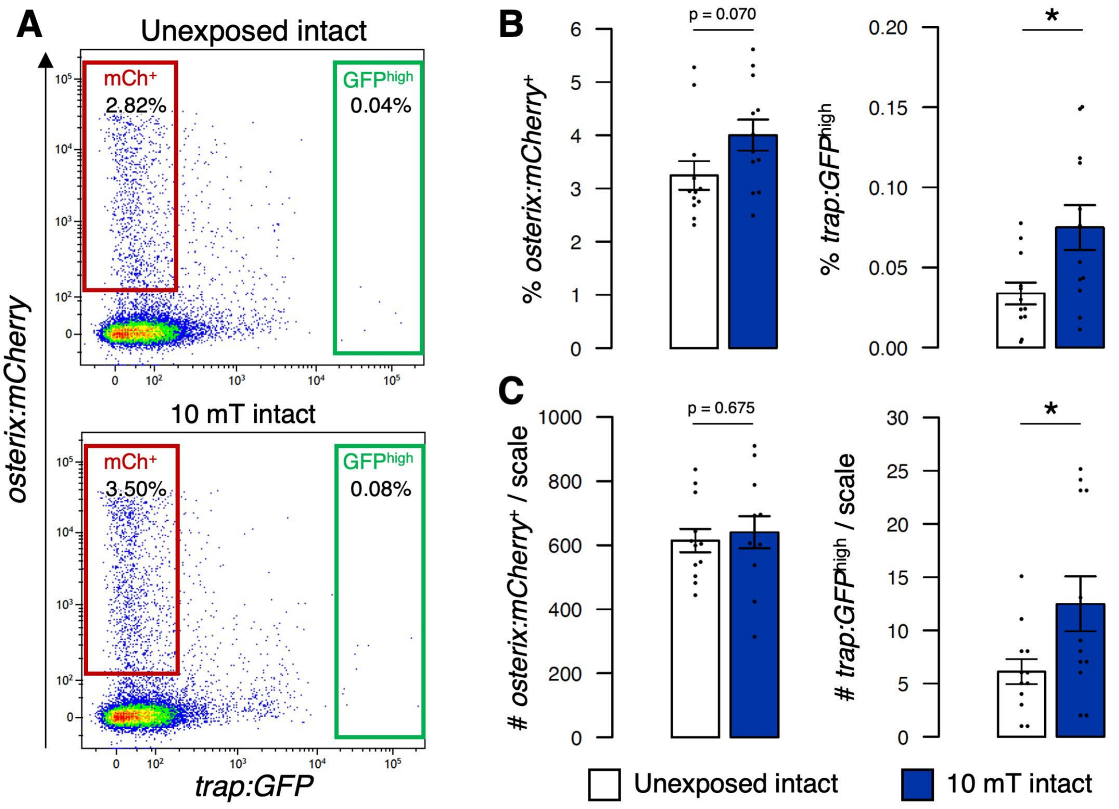

Supplementary Figure 3. Exposure to 10 mT ELF-EMFs increases osteoclasts in the zebrafish intact scale.

(**A**) Representative results of flow cytometric analysis of cells from intact scales unexposed or exposed to 10 mT ELF-EMFs. Red and green gates show *trap:GFP*$^{-}$ *osterix:mCherry*$^{+}$ ("mCh^{+}") and *trap:GFP*^{high} ("GFP^{high}") cells, respectively. (**B**, **C**) Percentage (**B**) and absolute number of mCh^{+} and GFP^{high} cells per zebrafish scale (**C**) in intact scales unexposed or exposed to 10 mT ELF-EMFs. A total of 12 samples from 3 zebrafish were used in each condition. Asterisks indicate the P-value in unpaired two-tailed Student's t-test. Error bars, s.e.m.; *$p < 0.05$.

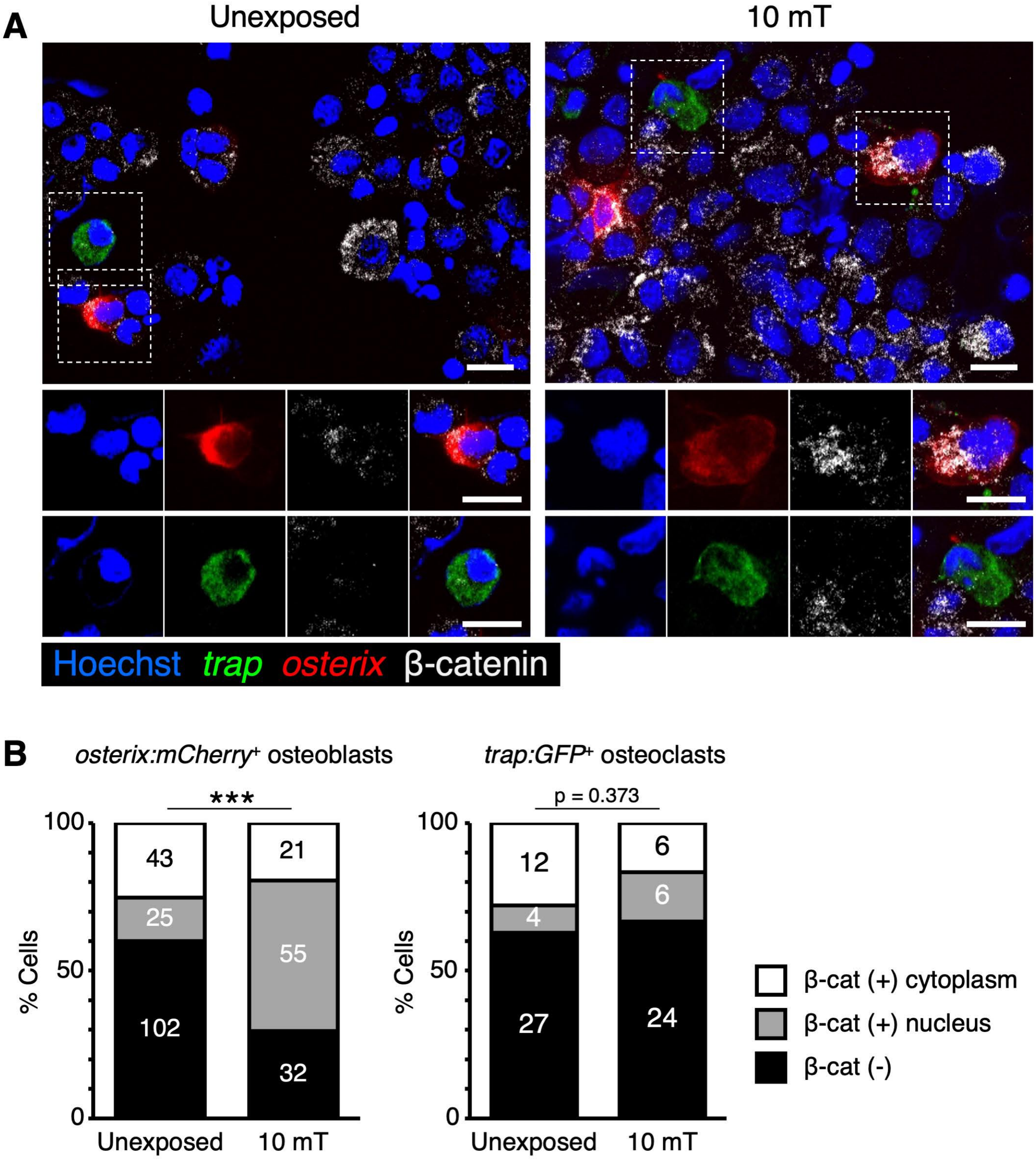

Supplementary Figure 4. β-catenin expression is enhanced in osteoblasts by exposure to 10 mT of ELF-EMFs.

(**A**) Expression of β-catenin in *osterix:mCherry*$^+$ osteoblasts or *trap:GFP*$^+$ osteoclasts from fractured scales unexposed or exposed to 10 mT ELF-EMFs at 4 h.p.fr. After fracture stimulation, zebrafish were placed in the ring-shaped tank and were unexposed or exposed to 10 mT ELF-EMFs for 4 h. Cells were then collected from zebrafish scales, smeared, and stained with rabbit anti-β-catenin antibody, followed by anti-rabbit IgG Alexa Fluor 647-conjugated secondary antibody and Hoechst 33342. Bottom panels show Hoechst (nuclei), *osterix:mCherry* or *trap:GFP* expression, β-catenin expression, and merged images of the dotted regions. Bars, 20 μm (upper panels); 10 μm (middle and lower panels). (**B**) Percent distribution of β-catenin expression in *osterix:mCherry*$^+$ osteoblasts or *trap:GFP*$^+$ osteoclasts. Cells were classified into three categories, β-catenin (+) only in the cytoplasm (β-cat (+) cytoplasm), β-catenin (+) in both the nucleus and cytoplasm (β-cat (+) nucleus), and β-catenin (-) (β-cat (-)). Total 170 and 108 osteoblasts unexposed and exposed to 10 mT, respectively, and 43 and 36 osteoclasts unexposed and exposed to 10 mT, respectively, from 3 zebrafish were analyzed. ***$p < 0.001$.

Supplementary Table 1. Primer sequences for qPCR

Gene	Forward primer	Reverse primer
ef1a	ACCGGCCATCTGATCTACAA	CAATGGTGATACCACGCTCA
ranlk	TAGTGTGGCGATTCTGTTGC	ATTGGAAGGTGAGCTGATGG
rank	AATCGCACGGTTATTGTTGTT	ACTGCAGCAAAGTCCCAGTT
opg	GTGAGTGTGAGGAGGGCTTC	CTGGCAGACTCGGTGTTTC
trap	ATGATGGCCAAAACTGCTTC	CAGCAATGACGTACCAAGGA
nfatc1	TCACTGCCTGCTCTTGATTG	CCTGGTAGAATGCGTGAGGT
alpl	GAGAAGCGGCCTGATTACTG	GTCTTAGAGAGGGCGACGTG
col1a1a	TTTTGGCAAGAGGACAAGGC	TGTCTTCGCAGATCACTTCG
wnt1	GTTAAAGCGGCATGTGTCCT	CGAGTACCAGCTGCACATTC
wnt3a	TCATGGCAAGCTACCCGATA	CTGATGCTGACACTCCTGGA
wnt10b	CAAGTTGCCCCATCAGAGTG	GTCATCCAGGCGTCGTTTAG
csf1a	AGCTGAGGAAGTGATTCGCA	TCCAATGGCTCGAGGATGAA
csf1b	TTTCTACAGGCTGGGCTTCA	CCGTTAGAAGGCTGTGGAGA
jag1a	ACGGAAGCGGATCTACTCCT	GTGTTTCAGGACCTGCCATT
jag1b	TGGTGAGCAAGCATAATGGA	GTGTTGCTGTGGGTGTTTTG
il1b	TTGTGGGAGACAGACAGTGC	CACTTCACGCTCTTGGATGA
dll4	ACGGGTCTTAACTGCGAGAG	TTTGCGCAGTCGTTAATGTT
fosl1b	TTTGTCACAGTGCTGCAGTC	GCGTCTTCACATGCTCCAAA
ccn1	CGTTACCTGCTTTCCGATCG	CAGACTTCTTGGTGCGGTTG
mmp9	AGACATTCGACGGAGACCTG	TCATGATGTCAGCAATGCCG

Supplementary Table 2. Primer and adaptor sequences for RNA-seq

Name	Sequence
RT-primer-001	CAGAAGACGGCATACGAGATcagcccaaGTGACTGGAGTTCAGACGTGTGCTCTTCCGATCTNNNNNNNNNTTTTTTTTTTTTTTTTTV
RT-primer-002	CAGAAGACGGCATACGAGATatgaatgtGTGACTGGAGTTCAGACGTGTGCTCTTCCGATCTNNNNNNNNNTTTTTTTTTTTTTTTTTV
RT-primer-003	CAGAAGACGGCATACGAGATtctagggaGTGACTGGAGTTCAGACGTGTGCTCTTCCGATCTNNNNNNNNNTTTTTTTTTTTTTTTTTV
RT-primer-004	CAGAAGACGGCATACGAGATttaattcaGTGACTGGAGTTCAGACGTGTGCTCTTCCGATCTNNNNNNNNNTTTTTTTTTTTTTTTTTV
RT-primer-005	CAGAAGACGGCATACGAGATtgagagcgGTGACTGGAGTTCAGACGTGTGCTCTTCCGATCTNNNNNNNNNTTTTTTTTTTTTTTTTTV
RT-primer-006	CAGAAGACGGCATACGAGATcgctgtgtGTGACTGGAGTTCAGACGTGTGCTCTTCCGATCTNNNNNNNNNTTTTTTTTTTTTTTTTTV
RT-primer-007	CAGAAGACGGCATACGAGATaacttttaGTGACTGGAGTTCAGACGTGTGCTCTTCCGATCTNNNNNNNNNTTTTTTTTTTTTTTTTTV
RT-primer-008	CAGAAGACGGCATACGAGATgcaccatgGTGACTGGAGTTCAGACGTGTGCTCTTCCGATCTNNNNNNNNNTTTTTTTTTTTTTTTTTV
RT-primer-009	CAGAAGACGGCATACGAGATgatacgtcGTGACTGGAGTTCAGACGTGTGCTCTTCCGATCTNNNNNNNNNTTTTTTTTTTTTTTTTTV
SE PCR-fw	CAAGCAGAAGACGGCATACGAGAT
SE PCR-rv	AATGATACGGCGACCACCGAGATCTACACACTCTTTCCCT
Adaptor-fw	A*C*C*GAGATCTACACACTCTTTCCCTACACGACGCTCTTCCGA*T*C*T
Adaptor-rv	/5Phos/G*A*T*CGGAAGAGCGTCGTGTTAAATGTA*T*A*T

* signifies a phosphonothioate bond; /5Phos/ signifies a phosphorylation.
